# Draft Genome Sequences of Endophytic Pseudomonas Strains, Isolated from the Interior of *Brassicaceae* Plants

**DOI:** 10.1128/mra.01337-22

**Published:** 2023-03-06

**Authors:** Hiroki Kaneko, Toshiki Furuya

**Affiliations:** a Department of Applied Biological Science, Faculty of Science and Technology, Tokyo University of Science, Chiba, Japan; University of Arizona

## Abstract

Members of the genus Pseudomonas have often been studied as agricultural biocontrol agents to activate plant immune responses. Here, we report the draft genome sequences of six Pseudomonas strains that were isolated from the interior of *Brassicaceae* plants.

## ANNOUNCEMENT

Microorganisms that colonize healthy plant tissues and activate plant immune responses are useful as agricultural biocontrol agents to reduce agrochemical use (1–3). Two endophytic strains belonging to the genus Pseudomonas, namely, BR1R-3 and BR1R-5, were recently isolated from the interior of Brassica rapa var. *perviridis* plants in Japan (4). These strains enhanced elicitor-induced reactive oxygen species (ROS) production in BY-2 tobacco cells, which can be monitored as a marker to evaluate the potential of microorganisms to activate plant immune responses (4). Additional Pseudomonas strains, i.e., RS1P-1, RS3R-1, RS3R-2, and RS3R-6, were isolated from another *Brassicaceae* plant, Raphanus sativus var. *hortensis*. These strains were also subjected to the assays to evaluate the potential of the microorganisms to activate plant immune responses (4). Interestingly, strains RS1P-1 and RS3R-1 enhanced elicitor-induced ROS production in BY-2 cells, whereas strains RS3R-2 and RS3R-6 did not exhibit this activity. The genomes of these six strains were sequenced to expand our understanding of the endophytic properties and plant immunity-activating potential of Pseudomonas strains.

The Pseudomonas strains examined in this study were cultured in NBRC802 medium, consisting of Hipolypepton (10 g/L), Bacto yeast extract (2 g/L), and MgSO_4_·7H_2_O (1 g/L) (pH 7.0). Single colonies were picked and inoculated into this medium for each strain. Bacteria were cultured at 30°C for 24 h (4). Bacterial genomic DNA was extracted using a NucleoBond high-molecular-weight (HMW) DNA kit (Macherey-Nagel, Düren, Germany) according to the manufacturer’s recommendations. All genomic DNA samples were sequenced by the Taniguchi Dental Clinic-Oral Microbiome Center (Kagawa, Japan), following the standard workflow for library preparation. For short-read sequencing, genomic libraries were prepared using a MGIEasy FS DNA library preparation set (MGI, Shenzhen, China), and sequencing was performed using a DNBSEQ-G400FAST sequencer and the DNBSEQ-G400RS high-throughput rapid sequencing set (2 × 150 bp; MGI). Read data and information are given in Table 1.

**TABLE 1:** Information and genome assembly statistics for the six endophytic Pseudomonas strains examined in this study

Characteristic	Data for strain:					
	BR1R-3	BR1R-5	RS1P-1	RS3R-1	RS3R-2	RS3R-6
Bacterial species	Pseudomonas atacamensis	Pseudomonas sp.	Pseudomonas moraviensis	Pseudomonas atacamensis	Pseudomonas lactis	Pseudomonas atacamensis
Isolate source	Brassica rapa var. *perviridis*	Brassica rapa var. *perviridis*	Raphanus sativus var. *hortensis*	Raphanus sativus var. *hortensis*	Raphanus sativus var. *hortensis*	Raphanus sativus var. *hortensis*
BioProject accession no.	PRJDB14730	PRJDB14730	PRJDB14766	PRJDB14766	PRJDB14766	PRJDB14766
BioSample accession no.	SAMD00554992	SAMD00554991	SAMD00556827	SAMD00557592	SAMD00557593	SAMD00557594
DRA accession no.	DRR417901	DRR417902	DRR417903	DRR417904	DRR417905	DRR417906
GenBank accession no. for contigs	BSCL01000001–BSCL01000056	BSCO01000001–BSCO01000086	BSCP01000001–BSCP01000060	BSCQ01000001–BSCQ01000080	BSCR01000001–BSCR01000085	BSCS01000001–BSCS01000080
No. of raw reads	31,743,508	35,120,183	5,888,087	37,465,822	37,368,968	30,784,310
No. of trimmed reads	2,223,737	2,108,041	2,310,980	2,248,516	2,242,720	2,156,593
Genome size (bp)	6,009,592	5,608,943	5,983,330	6,034,883	6,146,441	6,033,266
No. of contigs	56	86	60	80	85	80
No. of rRNAs	7	16	7	12	10	8
No. of tRNAs	66	86	66	70	68	70
*N*_50_ (bp)	376,337	446,660	528,396	499,410	363,124	499,410
Coverage (×)	111	111	113	111	110	107
Total no. of coding sequences	5,344	5,101	5,193	5,406	5,498	5,396
G+C content (%)	60.0	61.8	60.3	59.9	60.3	59.9

Reads were initially assessed using FastQC v0.11.9 (5). Adaptors and low-quality reads were then trimmed using fastp v0.20.1, and read pairs were collected from adapter-trimmed sequences using SeqKit v2.2.0 (6, 7). The trimmed reads were utilized for *de novo* assembly with Platanus_B v1.3.2 (8). Gene annotation was conducted using the Web application of DFAST v1.6.0 (https://dfast.ddbj.nig.ac.jp) (9). CheckM was used to perform completeness checks of the draft genome sequences in DFAST v1.6.0 (10). Taxonomic positions were determined using a digital DNA-DNA hybridization (dDDH) method (d4 formula) in the Type (Strain) Genome Server (TYGS) v342 (11, 12). Strains BR1R-3, RS3R-1, and RS3R-6 were assigned by TYGS to Pseudomonas atacamensis (dDDH values of 76.7%, 73.4%, and 73.4%, respectively, with respect to P. atacamensis M7D1^T^), strain RS1P-1 to Pseudomonas moraviensis (dDDH value of 82.4% with respect to P. moraviensis LMG 24280^T^), and strain RS3R-2 to Pseudomonas lactis (dDDH value of 88.2% with respect to P. lactis DSM 29167^T^). Strain BR1R-5 did not match any validly described species, with the closest type strain being Pseudomonas plecoglossicida DSM 15088 (dDDH value of 33.3%). Pseudomonas sp. strain BR1R-5 was suggested as a possible new species. The draft genomes were found to be 99.84% to 100.0% complete and to contain 0.08% to 0.54% contamination. All other genomic statistics are given in Table 1.

### Data availability.

The genome sequencing and assembly projects were deposited in the DDBJ under BioProject accession numbers PRJDB14730 and PRJDB14766. See Table 1 for BioSample accession numbers, DDBJ Sequence Read Archive (DRA) accession numbers, and GenBank accession numbers for contigs.

## References

[1] Liu H, Carvalhais LC, Crawford M, Singh E, Dennis PG, Pieterse CMJ, Schenk PM. 2017. Inner plant values: diversity, colonization and benefits from endophytic bacteria. Front Microbiol 8:2552. doi:10.3389/fmicb.2017.02552. 29312235 PMC5742157

[2] Reinhold-Hurek B, Hurek T. 2011. Living inside plants: bacterial endophytes. Curr Opin Plant Biol 14:435–443. doi:10.1016/j.pbi.2011.04.004. 21536480

[3] Van Wees SC, Van der Ent S, Pieterse CM. 2008. Plant immune responses triggered by beneficial microbes. Curr Opin Plant Biol 11:443–448. doi:10.1016/j.pbi.2008.05.005. 18585955

[4] Kurokawa M, Nakano M, Kitahata N, Kuchitsu K, Furuya T. 2021. An efficient direct screening system for microorganisms that activate plant immune responses based on plant-microbe interactions using cultured plant cells. Sci Rep 11:7396. doi:10.1038/s41598-021-86560-0. 33795728 PMC8016971

[5] Andrews S. 2010. FastQC: a quality control tool for high throughput sequence data. http://www.bioinformatics.babraham.ac.uk/projects/fastqc.

[6] Chen S, Zhou Y, Chen Y, Gu J. 2018. fastp: an ultra-fast all-in-one FASTQ preprocessor. Bioinformatics 34:i884–i890. doi:10.1093/bioinformatics/bty560. 30423086 PMC6129281

[7] Shen W, Le S, Li Y, Hu F. 2016. SeqKit: a cross-platform and ultrafast toolkit for FASTA/Q file manipulation. PLoS One 11:e0163962. doi:10.1371/journal.pone.0163962. 27706213 PMC5051824

[8] Kajitani R, Yoshimura D, Ogura Y, Gotoh Y, Hayashi T, Itoh T. 2020. Platanus_B: an accurate de novo assembler for bacterial genomes using an iterative error-removal process. DNA Res 27:dsaa014. doi:10.1093/dnares/dsaa014. 32658266 PMC7433917

[9] Tanizawa Y, Fujisawa T, Nakamura Y. 2018. DFAST: a flexible prokaryotic genome annotation pipeline for faster genome publication. Bioinformatics 34:1037–1039. doi:10.1093/bioinformatics/btx713. 29106469 PMC5860143

[10] Parks DH, Imelfort M, Skennerton CT, Hugenholtz P, Tyson GW. 2015. CheckM: assessing the quality of microbial genomes recovered from isolates, single cells, and metagenomes. Genome Res 25:1043–1055. doi:10.1101/gr.186072.114. 25977477 PMC4484387

[11] Auch AF, von Jan M, Klenk HP, Göker M. 2010. Digital DNA-DNA hybridization for microbial species delineation by means of genome-to-genome sequence comparison. Stand Genomic Sci 2:117–134. doi:10.4056/sigs.531120. 21304684 PMC3035253

[12] Meier-Kolthoff JP, Göker M. 2019. TYGS is an automated high-throughput platform for state-of-the-art genome-based taxonomy. Nat Commun 10:2182. doi:10.1038/s41467-019-10210-3. 31097708 PMC6522516

